# Experimental Investigation on the Breaching Process of Landslide Dams with Differing Materials under Different Inflow Conditions

**DOI:** 10.3390/ma15062029

**Published:** 2022-03-09

**Authors:** Zhenming Shi, Gongding Zhang, Ming Peng, Qingzhao Zhang, Yuanyuan Zhou, Mingjun Zhou

**Affiliations:** 1 Key Laboratory of Geotechnical and Underground Engineering of Ministry of Education, Department of Geotechnical Engineering, Tongji University, Shanghai 200092, China; 94026@tongji.edu.cn (Z.S.); 1810039@tongji.edu.cn (G.Z.); zhangqingzhao@tongji.edu.cn (Q.Z.); yuanyuan_zhou@tongji.edu.cn (Y.Z.); mjzhou@tongji.edu.cn (M.Z.); 2 Department of Geotechnical Engineering, College of Civil Engineering, Tongji University, Shanghai 200092, China

**Keywords:** landslide dam, dam material, breaching process, outflow discharge, erosion resistance

## Abstract

Landslide dams are dangerous because the outburst floods produced by dam failures seriously threaten life and property downstream. In this study, a series of physical flume tests were conducted to investigate the breaching process of landslide dams with fine-grained, well graded, and coarse-grained material under different inflow conditions. The effects of dam material and inflow discharge on the breach development, outflow discharge and erosion characteristics were studied. The erosion resistance of materials and lateral collapses were also discussed. Experimental results reveal that the whole breaching process is determined by the water-sediment interaction. For the fine-grained dams, a general constant downstream slope angle is maintained during the breaching process. For the well-graded dams, a step-pool structure is generated due to the scarp erosion. For the coarse-grained dams, they can remain stable under normal circumstances but fail by overtopping in a short duration under the extreme inflow condition. The final breach of the dam with higher fine content or larger inflow discharge is deeper and narrower. In addition, many fluctuations are observed in the changing curve of the erosion rates along the flow direction for the well-graded and coarse-grained dams. The erosion resistance of materials increases along the flow direction, which needs to be further considered in physically based breach models. Furthermore, the lateral collapse is affected by the dam material instead of inflow discharge. The lower fine content causes more lateral collapses with smaller volumes.

## 1. Introduction

Landslide dams are natural deposits formed by river blockages from landslides, collapses, debris flows, and so on [1,2]. Most landslide dams are widely distributed in mountainous areas worldwide because their required conditions are not difficult to attain [3]. As a type of natural hazard, landslide dams frequently block rivers and form barrier lakes [4]. Many cases in the literature indicated that the outburst floods induced by landslide dam failures presented serious catastrophes to human lives and properties downstream. For instance, in 1933, the Diexi landslide dam failed by overtopping; the outburst flood washed away a large number of villages and farmlands along the river and led to more than 2500 fatalities [5,6]. In 2008, the Tangjiashan landslide dam, induced by the Ms 8.0 Wenchuan earthquake, formed a barrier lake with a total volume of 316 million m^3^, threatening more than 1.3 million people in downstream areas [7]. From the aforementioned devastating consequences of landslide dam failures, it is very crucial to study the breaching process and mechanisms of landslide dams.

The breaching process of manmade dams has attracted numerous attentions of scholars [8,9,10]. Compared with manmade dams, landslide dams are formed by an unconsolidated mixture of earth or rock debris and the granular material ranges a wide grain size distribution [2,11]. In addition, various triggers and forming locations result in differing grain compositions of different landslide dams, which may greatly affect the dam failures [12]. Therefore, the breaching characteristics of landslide dams are significantly differed from manmade dams. Previous studies on landslide dam failures mainly contain four parts: case investigations, statistical analyses, numerical simulations, and model experiments [4,13,14,15,16,17,18,19].

In case investigations, the failure mode of landslide dams was considerably related to the dam material [20,21]. Overtopping was the primary failure mode of landslide dams [22,23]. The breaching durations of landslide dams induced by the *M_s_* 8.0 Wenchuan earthquake were affected by the grain compositions and sedimentologic structures [24,25]. Case investigation work is meaningful for understanding the disaster process of a specific landslide dam case. However, such work is not enough for detail research about the influence of dam material on the failure mode and breaching duration of landslide dams.

In statistical analyses, Costa and Schuster (1991) studied the effects of dam material on the outburst flood based on a database of 225 landslide dam cases [1]. Peng and Zhang (2012) presented an empirical model considering the dam morphology and material erodibility for estimating several breaching parameters (e.g., peak outflow discharge and breaching duration) based on a database of 1239 landslide dam cases [12]. These studies attempt to establish a rapid method to forecast the breaching parameters of landslide dams, but they cannot predict the detailed changes in breach geometry with time, especially for abruptly occurring landslide dams with limited information.

In numerical simulations, a shallow water model was popularly used to simulate the dam-break flows and sediment transports [26,27,28]. Pu et al. (2013) improved the standard shallow water equations (SWEs) model using Surface Gradient Upwind Method (SGUM) to compute dam-break flows and compared the performance of the SGUM-SWEs model and SPH model [27]. Through the numerical approach, detailed flow information (e.g., water depth, flow velocity, flow surface profile) can be obtained accurately, and the computational time is relatively short. Additionally, several physically based mathematical models were established to simulate the breach development of landslide dams for practical application [29,30,31,32]. Chang and Zhang (2010) presented a breach model considering the variation in the soil erodibility along depth [33]. Zhong et al. (2018) presented a breach model by revising the distribution of erosion rate on the downstream slope of the dam along the flow direction [34]. Dam material parameters such as the soil erodibility are considered as one of the most important intrinsic factors which governing the erosion process in existing models. Nevertheless, the distribution of erosion rate along the flow direction and the erosion resistance at different cross-sections of the dam, which considerably affect the accuracy of simulation results, have not been adequately explored.

In model experiments, some researchers studied the influence of dam material on the failure mode and outflow discharge of landslide dams by flume tests. Gregoretti et al. (2010) carried out laboratory experiments by using three types of dam material whose grain sizes varied in the ranges of 2–5, 5–9.5, and 6–13 mm. Three main typologies of dam failure were observed: (1) overtopping, (2) headcutting, and (3) superficial slide [35]. Cao et al. (2011) experimentally studied the effects of non-cohesive sand, cohesive clay, and gravel on the outburst flood and noted that the cohesive clay might strengthen the flood, and the gravel might significantly depress the flood [36]. Chen et al. (2015) carried out flume tests by using two types of dam material (earthquake- and rainfall-induced) and claimed that the dam with the looser material had a larger peak outflow discharge compared with the denser material [37]. Jiang et al. (2018) analyzed the effects of median diameter and fine content of materials on the outburst flood through seven flume tests and indicated that the peak outflow discharge decreased with the increase of the median diameter [38]. Zhu et al. (2020) experimentally studied the relationship between the failure mode and dam material and summarized five failure types of landslide dams (including the stable status) [39]. The experimental studies substantially contributed to the study of landslide dam breaching mechanisms. However, the influence of dam material on the breaching process was usually studied in a specific inflow rate. The lack of analyses on dam material effects under different inflow discharges leaves the study incomplete, especially considering the failure of a natural landslide dam is significantly affected by distinctive geological and inflow conditions. Additionally, further investigation is necessary to figure out the influence of dam material and inflow discharge on the erosion characteristics and lateral collapse.

This study presents the experimental results of landslide dam breaching. These experiments were designed to (i) determine the failure modes and breaching characteristics of landslide dams with differing grain compositions, especially under different inflow conditions; (ii) identify the relationship between erosion rate and dam material during the breaching process; and (iii) investigate whether the material and inflow discharge have significant effects on the lateral collapse. The structure of the paper is as follows. Firstly, the experimental flume setup, model dam geometry, dam material type, and experimental design are described. Then, the breaching processes of landslide dams with differing grain compositions under different inflow discharges are compared, and the failure modes of dams are discerned. Then, the effects of dam material and inflow discharge on the breach development and outflow discharge are investigated. Finally, the distribution of erosion rate along the flow direction, the erosion resistance of dam materials, and the differences between lateral collapses of landslide dams with differing grain compositions are discussed, respectively.

## 2. Flume Tests on Landslide Dams

### 2.1. Experimental Setup

**Figure 1:**
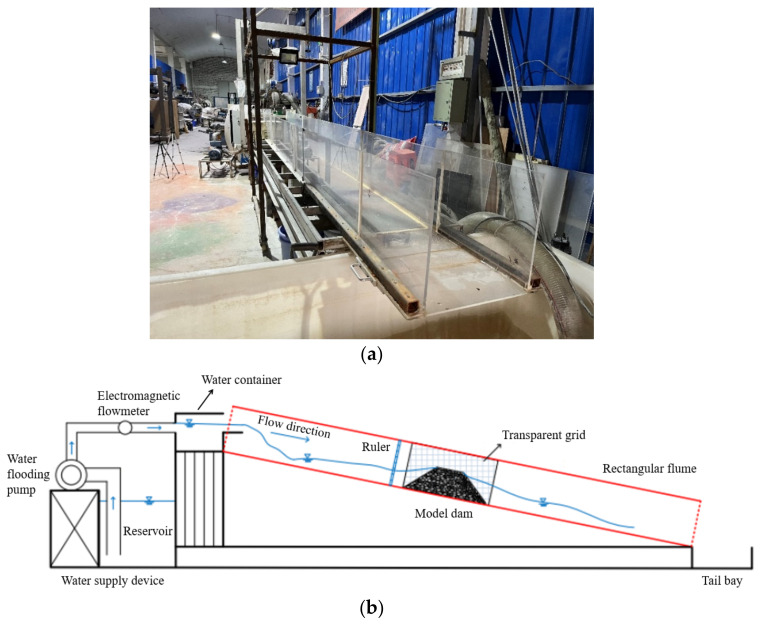
Experimental apparatus: (**a**) a front view of the flume viewed from the downstream region; (**b**) schematic diagram of the flume system [41].

All the experiments were conducted in the flume system, as shown in Figure 1. The flume system consisted of three parts: a flow supply device, a rectangular flume, and a tail bay. The flow supply device contained a reservoir with a capacity of 6 m^3^ (*l* × *w* × *h* = 2 m × 2 m × 1.5 m), a water flooding pump with a maximum inflow rate of 3.0 L/s, and an electromagnetic flowmeter with an accuracy of 0.01 L/s. The inflow was supplied at a constant flow rate during each test. The rectangular flume had a length of 5.0 m, a width of 0.4 m, and a height of 0.4 m. The flume sidewalls were made of transparent acrylic sheets, allowing the breaching process of landslide dams to be observed clearly. The flume bottom slope was fixed at 1°, considering that the riverbed slope of a natural landslide dam is in the range of 0–3° [40]. A water container with a capacity of 32 L was connected to the top of the flume through a saw-teeth eliminator, which effectively minimized the splashing waves from the water flooding pump. The tail bay with a capacity of 1 m^3^ (*l* × *w* × *h* = 2 m × 1 m × 0.5 m) was arranged at the end of the flume to collect the water and sediments from the channel.

### 2.2. Model Dam

**Figure 2:**
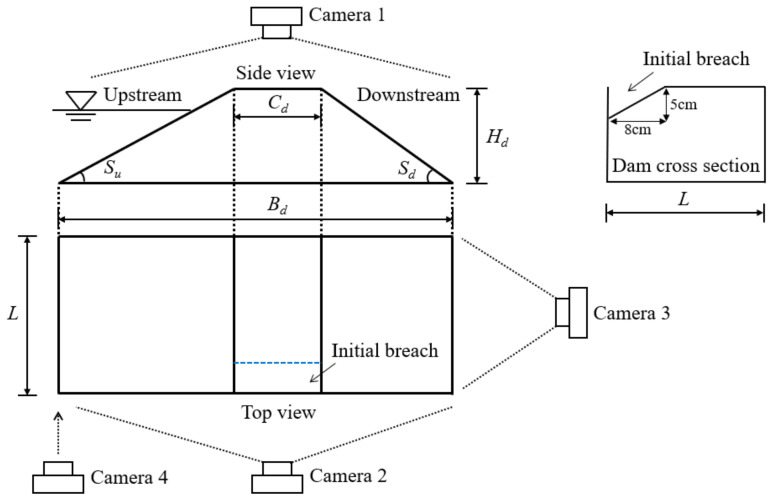
Model dam and measurements [41].

The dam with a trapezoid longitudinal section was simulated in the experiments, as shown in Figure 2. The dam height *H_d_* was 0.24 m, considering that the flume height was 0.40 m. The upstream and downstream slopes of the dam were 1:2 (*S_u_* = 26.6°) and 1:1.5 (*S_d_* = 33.7°), respectively, because the slope of a natural landslide dam is between 11–45° [42]. The dam crest width *C_d_* along the flow direction was 0.24 m and the dam bottom width *B_d_* was 1.08 m, in light of the ratio of dam height to dam crest width of a natural landslide dam ranges from 0.2 to 3.0 [33]. The transversal section of the dam was a rectangle, and the dam length *L* was 0.40 m to match the flume width, seen in Figure 2. A triangular initial breach (depth × width = 5 cm × 8 cm) was excavated on the dam crest to simulate the artificial spillway constructed in a natural landslide dam (Figure 2). The initial breach was adjacent to the flume sidewall, allowing the breach dimension and the water depth in the breach to be captured accurately. The dam was located 2.1 m away from the front section of the flume, considering that the dam region constituted the central section of the channel.

Geometric scaling laws must be satisfied in designing physical model tests to ensure that the model dam is able to represent natural landslide dams. Herein, three important geometrical parameters of landslide dams ($H_{d}/B_{d}$, $V_{d}^{1/3}/H_{d}$, and $V_{l}^{1/3}/H_{d}$) were carefully considered [12], as shown in Table 1. The three above dimensionless numbers set in the experiments fall within the reasonable range of values based on a database of 80 landslide dam cases [19], verifying that the model dam in this study could simulate real large-scale landslide dams. In addition, there are some other real-world factors that may cause discrepancy for the experiments, such as the measurement method and condition [43], the irregular or compound channel shape [44], and the real-world water-worked bed condition [45,46]. This study aims to obtain some preliminary results of the breaching characteristics of landslide dams with different grain compositions. Due to this, the cross-section of the flume was set as a rectangle for simplicity (Figure 1a). A rigid riverbed was used in the downstream area of the flume. The rigid riverbed had no sediment to simulate a rock riverbed [37,47].

### 2.3. Dam Material

**Figure 3:**
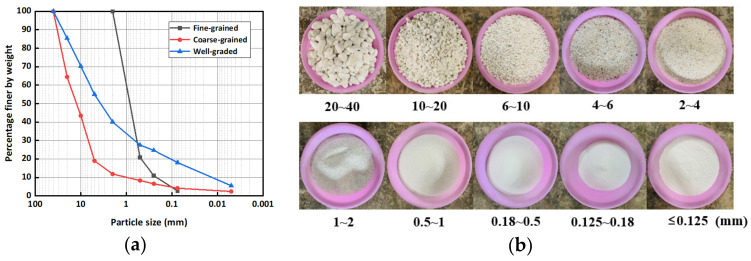
Experimental materials: (**a**) grading curves of landslide dam materials; (**b**) pebbles and silica sands used in the experiments [41].

The materials of natural landslide dams have a wide grain size distribution. Therefore, proper selection of the grain composition of the dam is critical for physical model tests. As shown in Figure 3a, three typical grain compositions were derived from the Donghekou, Tangjiashan, and Xiaogangjian landslide dams induced by the Wenchuan earthquake [33,48]. The Donghekou landslide dam was caused by a typical rapid, long runout, compound landslide [49,50]. It was mainly composed of sandstone, shale, and schist of Cambrian age [49,51], which disintegrated into relatively small particles along the movement paths [52]. As the largest and most dangerous landslide dam induced by the Wenchuan earthquake, the Tangjiashan landslide dam was caused by a massive rockslide in interbedded soft rock and hard rock strata [53,54]. It consisted of medium highly weathered schist, slate, and sandstone dipping parallel to the slope [49]. The Xiaogangjian landslide dam was caused by a shattering-sliding type of landslide consisting of dolomite interbedded with dolomitic limestone [33], which contained much more coarse grains than the above two landslide dams. In this study, the grading curve that represented the average level was selected from the sieving test results to prepare the model dam [47], making the experimental grading similar to that of natural landslide dam [55]. A similar method has been adopted by numerous researchers [14,56,57]. The three grading curves represented (1) fine-grained (the Donghekou landslide dam), (2) well-graded (the Tangjiashan landslide dam), and (3) coarse-grained (the Xiaogangjian landslide dam) material, respectively. The three above debris types were defined based on landslide dam materials instead of the standard engineering classification of soils. The median diameters *d*_50_ of the fine-grained and well-graded materials were 0.8 mm and 3.8 mm, respectively, which were smaller than the value of the coarse-grained material (13.6 mm), as shown in Table 2. In addition, the fine content *p* of the fine-grained and well-graded materials were 50.2% and 33.5%, respectively, which were larger than the value of the coarse-grained material (10.3%).

The dry density *ρ_d_* of the dam in the experiments was determined to be 1780 kg/m^3^, which was similar to the drillhole data of natural landslide dams [21,33,42]. The model dam was prepared by mixing pebbles and quartz sands in different proportions. The pebbles and quartz sands were sieved into 10 different ranges of grain size: 20–40, 10–20, 6–10, 4–6, 2–4, 1–2, 0.5–1, 0.18–0.5, 0.125–0.18, and ≤0.125 mm, as shown in Figure 3b. Firstly, the percentage content of each granular group was determined according to the required grain composition (Figure 3a). Secondly, the weights of different groups were obtained after the total mass of the dam was calculated. Finally, the granular groups were adequately mixed by repeated stirring to make the dam materials homogeneous.

The relevant parameters varied in the experiments were the dam material and inflow discharge, as shown in Table 2. All the tests in this study could be divided into three groups according to the dam material type: fine-grained dams (Tests 1–3), well-graded dams (Tests 4–6), and coarse-grained dams (Tests 7–10). Different inflow discharges (*Q_in_* = 0.5, 0.75, 1.0 and additional 2.0 L/s) were tested in each group, based on the geometric scaling and Froude scaling of the inflow rate of natural landslide dams.

### 2.4. Measurements

The experimental processes, including the dam status, breach development, and water level variation, were recorded by four digital cameras at different positions, as show in Figure 2. Camera #1 combined with a high precision steel ruler (minimum scale = 1 mm) was installed above the dam crest to measure the breach top and bottom width. The breach top width could be captured directly, and the breach bottom width was simplified to be equal to the width of water flow in the breach [58]. A transparent grid (the length of a single grid was 2 cm) was pasted on the glass panel of the flume side, and Camera #2 was positioned at the side of glass panel to record the longitudinal evolution of dams. The variations of the breach depth and water depth were also captured by Camera #2, and the measured water depth was the distance between the flow surface and the breach bottom. Camera #3 was positioned at the end of the flume to record the transversal evolution of dams. The downstream slope status, lateral collapses as well as the breach shape viewed from the downstream region were also captured by Camera #3. Camera #4 combined with a steel ruler (minimum scale = 1 mm) was installed at the side of the barrier lake to measure the upstream water level. All the cameras used in the experiments were GZ-R10BAC (1920 × 1080 pixel; JVC, Yokohama, Japan), which could provide high quality experimental videos.

**Figure 4:**
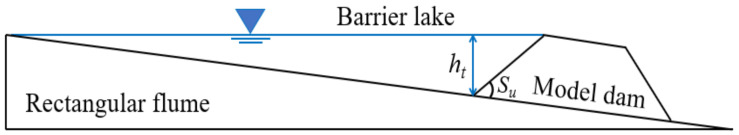
Schematic diagram of the barrier lake and model dam.

The outflow discharge *Q_out_* was accurately calculated from the upstream water level *h_t_*, as shown in Figure 4. When the variation of the upstream water level in real time was captured by camera #4, the capacity of the barrier lake *V_l_* at different times was determined. Then, the water balance equation for the barrier lake was applied to calculate the outflow discharge *Q_out_* during the breaching process [59]:

(1) $$Q_{in}-Q_{out}=\frac{dV_{l}}{dt}$$

where *Q_in_* is the inflow discharge, *Q_out_* is the outflow discharge after dam failure, *V_l_* is the capacity of the barrier lake, and *t* is the time.

### 2.5. Experimental Procedure

The detailed procedure in the experiments was as follows:

(1) Experimental preparation. The dam outline and transparent grid were pasted on the flume sidewall according to the geometry of the predesigned dam.
(2) Dam construction. The model dam was built in three layers based on the contour line with 8 cm thick in each floor by using a density control method (*ρ_d_* = 1780 kg/m^3^). Every layer was fully compacted by slightly tapping to obtain the required dry density. After the model dam was constructed, the dam crest surface was carefully smoothed and leveled, and then, an initial breach was excavated adjacent to the flume sidewall.
(3) Data capture. The digital cameras and steel rulers were installed at their specified positions. The videos and images collected by the four cameras were autosaved on a computer during each test.
(4) Water inpouring. The water flooding pump operated after the reservoir was full of water. The inflow discharge was maintained at the predesigned rate during each test by means of the electromagnetic flowmeter.
(5) Dam processing. After the dam either absolutely failed or remained stable for more than 1 h, the test was considered to be terminated. The flow supply device and cameras were stopped. The residual dam from the channel was fully removed, and a new model dam was constructed for the next test.

## 3. Experimental Results

In the succeeding discussions, the initial time of the breaching process *t_0_* = 0 s denoted the moment when the drainage water passed through the entire initial breach and eventually reached the downstream crest of the dam.

### 3.1. General Features

#### 3.1.1. Fine-Grained Dams

**Figure 5:**
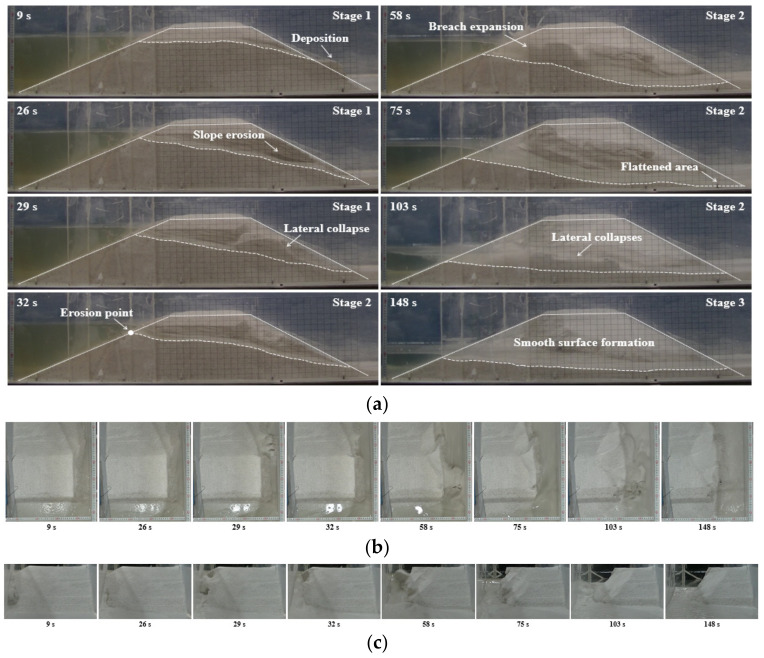
Breaching process of Test 1: (**a**) photographs from the side perspective; (**b**) photographs from the top perspective; (**c**) photographs from the downstream.

For the dam with the fine-grained material in Test 1 (*Q_in_* = 0.5 L/s), the breaching process was shown in Figure 5. A discernable erosion gully was initially formed on the downstream slope surface after the overflow exited the initial breach (see *t* = 9 s in Figure 5). The overflow was insufficient to cause an extensive erosion to dam due to its shallow depth, small velocity, and limited quantity. Therefore, most eroded materials within the developing gully were simply deposited on the dam toe (see *t* = 9 s in Figure 5). Next, the gully got deepened and its side slope was gradually eroded (see *t* = 26 s in Figure 5). Subsequently, a lateral collapse at the middle of the downstream slope occurred, causing the gully herein to broaden instantaneously (see *t* = 29 s in Figure 5). At *t* = 32 s, the dam crest was completely eroded, and the dam longitudinal section became triangular in shape (Figure 5), which meant that the headward erosion significantly developed besides the vertical and lateral erosions. By this point, the breach transversal shape approached to a rectangle (see *t* = 32 s in Figure 5). After reaching its maximum, the upstream water level began to drop as the dam height at the upstream crest decreased. The continuously increasing overflow caused the breach entrance to incise and expand (see *t* = 58 s in Figure 5), allowing a larger amount of water to be released downstream. In this scenario, the water depth in the breach rapidly increased, and the erosion process was obviously accelerated. At *t* = 75 s, the outflow discharge reached the peak (*Q_p_* = 2.86 L/s). Many grains were flushed downstream by torrential flood, and the downstream slope of the dam was almost flattened (see *t* = 75 s in Figure 5). Then, the outflow discharge started to attenuate, and the sloping breach channel gradually flattened, which meant that the vertical erosion steadily weakened, and the lateral erosion became the predominant erosion mechanism. Three lateral collapses with volumes larger than before continuously occurred and temporarily blocked the breach channel (see *t* = 103 s in Figure 5). The outflow stopped until the blockages were scattered and pushed away. Herein, the breach transversal shape changed from a rectangle to a trapezoid (see *t* = 103 s in Figure 5). Afterwards, the elevation of the breach entrance almost no longer decreased, and the erosion balance was achieved (see *t* = 148 s in Figure 5). Finally, the breach reached its final dimensions, and the outflow discharge was asymptotically close to the inflow discharge. The residual dam with a smooth surface was produced, and the breaching process was terminated. For the dams with the fine-grained material in Test 2 (*Q_in_* = 0.75 L/s) and Test 3 (*Q_in_* = 1.0 L/s), when the inflow discharge increased, the failure speed was accelerated, and the breaching duration decreased. Additionally, the peak outflow discharge increased with an increase in the inflow discharge. More details about the effects of inflow discharge can be seen in Section 3.3 and Section 3.4.

#### 3.1.2. Well-Graded Dams

**Figure 6:**
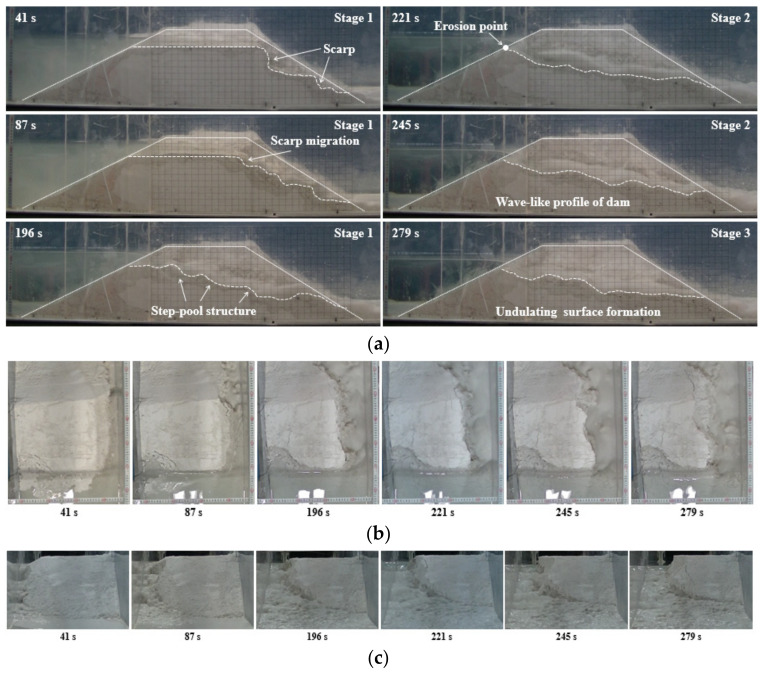
Breaching process of Test 4: (**a**) photographs from the side perspective; (**b**) photographs from the top perspective; (**c**) photographs from the downstream.

For the dam with the well-graded material in Test 4 (*Q_in_* = 0.5 L/s), the breaching process was presented in Figure 6. The scarp similar to a waterfall in geomorphologic feature was initially formed on the downstream slope surface (see *t* = 41 s in Figure 6) because the well-graded material had a larger shear strength and hence was more difficult to be transported compared with the fine-grained material. Next, the scarp bottom was gradually eroded by the overflow, causing some coarse grains upper the scarp to slide slowly. Subsequently, the scarp collapsed and began to head towards the upstream slope (see *t* = 87 s in Figure 6). A step-pool structure was generated on the dam longitudinal profile as the scarp continuously moved (see *t* = 196 s in Figure 6). Meanwhile, quite a number of lateral collapses with small volumes occurred frequently. At *t* = 221 s, the dam crest was completely eroded (Figure 6), and this duration from the beginning of breaching process to the erosion of upstream crest (221 s) was much longer than the value of Test 1 (32 s) due to the scarp migration. Then, the outflow discharge increased until reaching the peak (*Q_p_* = 1.21 L/s), and the dam longitudinal profile was scoured in a wave-like type (see *t* = 245 s in Figure 6). Afterwards, as the outflow discharge decreased, an armored layer was gradually formed on the breach channel surface due to the existence of coarse sediments (see *t* = 279 s in Figure 6). The armored layer could protect the sediments from being further transported, contributing to the formation of a stable residual dam. In this scenario, the surface of the residual dam was rough and undulating (see Figure 6). Eventually, the residual dam height for Test 4 (12.6 cm) was much larger than the value of Test 1 (3.7 cm). For the dams with the well-graded material in Test 5 (*Q_in_* = 0.75 L/s) and Test 6 (*Q_in_* = 1.0 L/s), with the increasing of the inflow discharge, the scarp height and volume significantly increased, and the speed of the scarp migration was substantially accelerated. Additionally, it should be noted that the residual dam height considerably decreased with the increasing inflow discharge. More details about the effects of inflow discharge referred to Section 3.3 and Section 3.4.

#### 3.1.3. Coarse-Grained Dams

**Figure 7:**
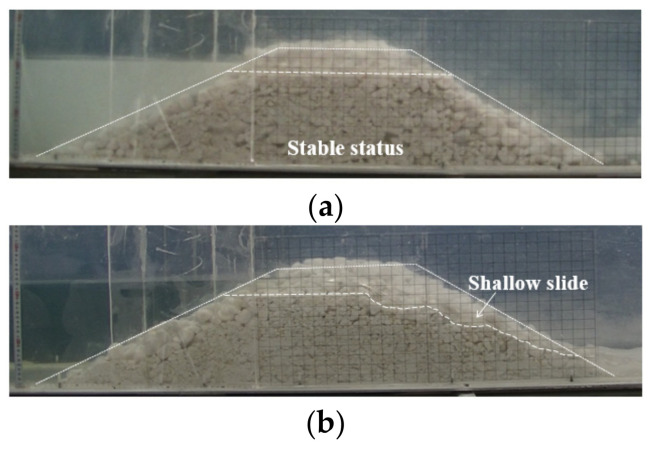
Longitudinal snapshots of the coarse-grained dams: (**a**) Tests 7–8; (**b**) Test 9.

For the dams with the coarse-grained material in Test 7 (*Q_in_* = 0.5 L/s) and Test 8 (*Q_in_* = 0.75 L/s), the landslide dams did not fail, and remained stable for more than 1 h, as shown in Figure 7a. The considerable seepage was initially observed on the downstream slope before overflowing. The larger seepage flow can be observed with higher upstream water level. Most finer grains below the seepage line were transported downstream, and the seepage flow was muddy. Nevertheless, the seepage-induced transport was too weak to loosen dam structure and remove coarser grains. After the drainage water overflowed, the initial breach and the initial breach did not develop, which meant that the outflow did not have enough energy to erode the coarse-grained materials. Eventually, the upstream water level remained unchanged, and the outflow water gradually cleared. For the dam with the coarse-grained material in Test 9 (*Q_in_* = 1.0 L/s), when the inflow discharge increased, a shallow slide near the downstream crest of the dam occurred after overflowing, as shown in Figure 7b. The initial breach got deepened and broadened by the slide, subsequently magnifying the outflow discharge. Afterwards, the inflow–outflow reached balance, and the dam was restored to stable status again. All the above experimental results reflected the extremely stable structure of the coarse-grained material dam.

**Figure 8:**
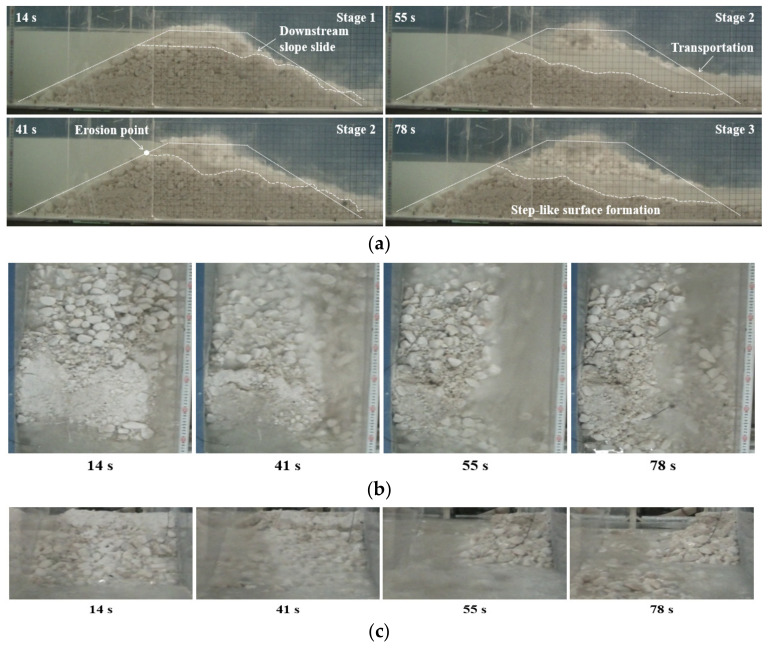
Breaching process of Test 10: (**a**) photographs from the side perspective; (**b**) photographs from the top perspective; (**c**) photographs from the downstream.

As the dam with the coarse-grained material did not fail when *Q_in_* ≤ 1.0 L/s, the inflow discharge was doubled to 2.0 L/s to trigger the dam breaching in Test 10. The breaching process under the extreme inflow condition was shown in Figure 8. The downstream slope initially slid with the rapid increasing upstream water level (see *t* = 14 s in Figure 8). Next, the breach got further deepened and broadened because the shear stress induced by the overflow was large enough to wash away the coarse grains. In this condition, the dam crest was completely eroded in a short period (see *t* = 41 s in Figure 8), which was similar to the fine-grained material dam. Then, as the water depth in the breach sharply increased, the dam body slide intermittently occurred, and a large number of grains with various sizes was transported downstream together like a debris flow (see *t* = 55 s in Figure 8). Afterwards, the erosion process attenuated as the upstream water level rapidly decreased. Eventually, the residual dam with a step-like surface was produced (see *t* = 78 s in Figure 8).

### 3.2. Comparison of the Failure Modes

**Figure 9:**
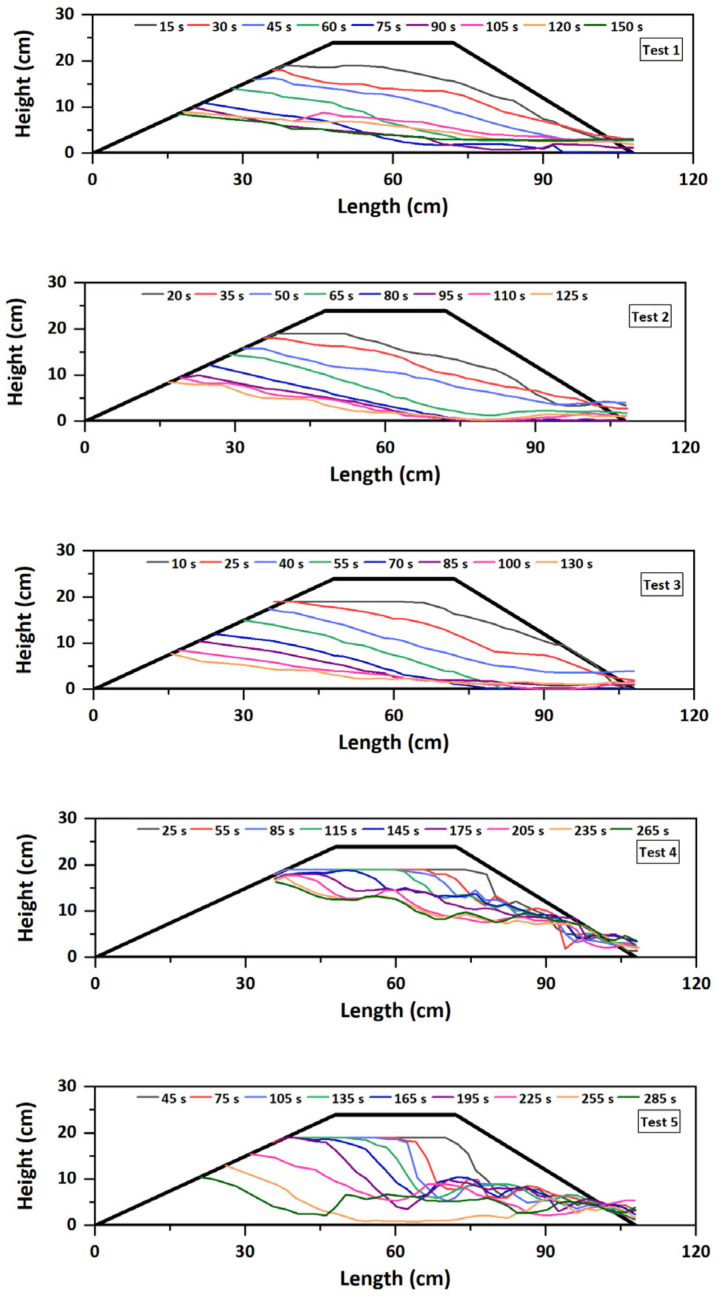

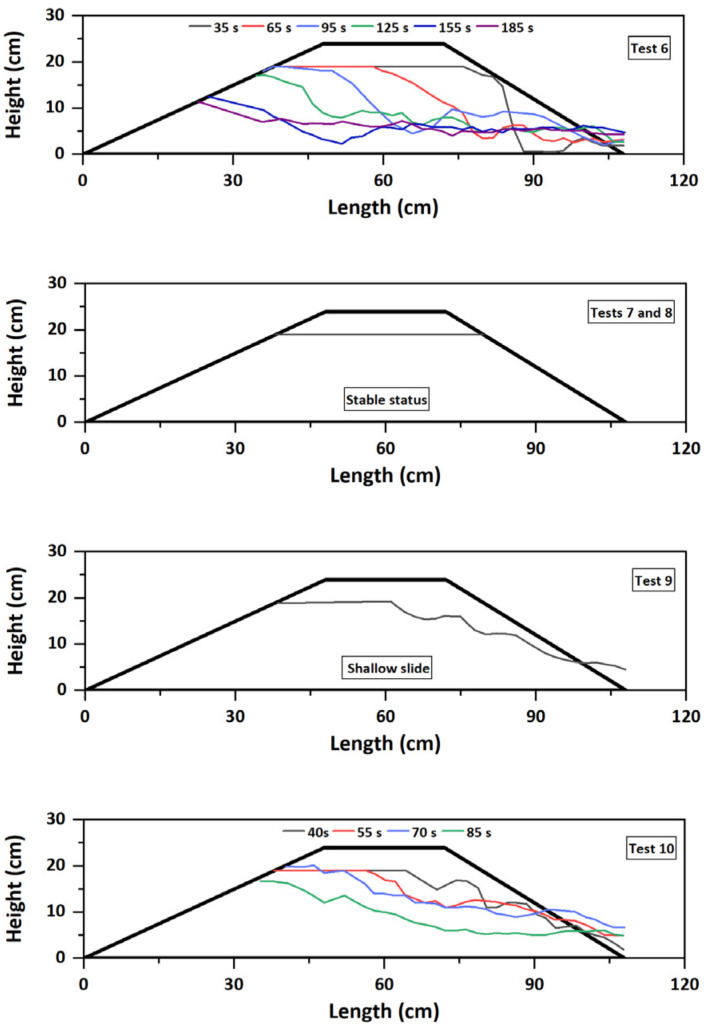
Evolution of dam longitudinal profiles.

The evolutions of the dam longitudinal section during the breaching process were shown in Figure 9. The breaching processes of landslide dams with different grain compositions obviously differed. For the fine-grained dams (Tests 1–3), the dam materials were very easy to be washed away; the dams all failed by overtopping, and the breach channel approximately retreated in parallel during the breaching process. The downstream slope angle decreased rapidly at first and then stayed at a relatively constant angle in the range of 11.2–14.1°. For the well-graded dams (Tests 4–6), a step-pool structure was generated on the dam longitudinal profile after overflowing due to the scarp scour. The downstream slope angle drastically changed during the whole breaching process. Finally, the formation of an armored layer made the sediments undisturbed, and the residual dam surface undulating. For the coarse-grained dams (Tests 7–10), when *Q_in_* ≤ 1.0 L/s, the dam remained stable (Tests 7–8) or restored overall stability after the occurrence of a shallow slide (Test 9). When *Q_in_* = 2.0 L/s, the overtopping failure rapidly developed, and the instantaneous slip of the dam body was triggered (Test 10).

**Figure 10:**
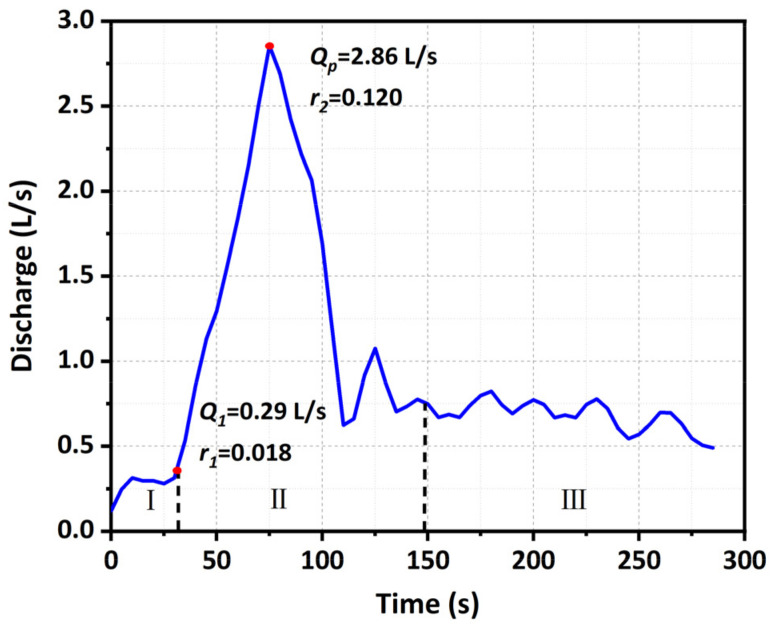
Outflow discharge hydrograph of Test 1. *Q*_1_ is the final discharge prior to Stage II; *Q_p_* is the peak discharge; *t*_1_ is the duration of Stage I; *t_p_* is the arrival time of the peak; *r*_1_ is the dimensionless changing rate of discharge in Stage I ($\frac{Q_{1}}{Q_{in\;}t_{1}}$); *r*_2_ is the dimensionless changing rate of discharge in Stage II ($\frac{Q_{p}-Q_{1}}{Q_{in}\;\left(t_{p}-t_{1}\right)}$ ).

As the breaching characteristics of landslide dams with different grain compositions were distinct, clearly defining and dividing the stages of the breaching process could facilitate the quantitative comparison of different tests. Based on the dam longitudinal section evolution (Figure 9) and outflow discharge hydrograph (Figure 10), the breaching process was generalized as three distinct stages (taking Test 1 as an example). Stage I (the breach initiation stage) started from the moment when the overflow was released from the downstream crest of the dam. During Stage I, the water depth in the breach was shallow, and the flow velocity was small, which resulted in slow development of the breach. Both the outflow discharge and its changing rate were small (*Q*_1_ = 0.29 L/s, *r*_1_ = 0.018), as shown in Figure 10. Stage I ended when the dam crest was completely eroded. During Stage II (the breach development stage), the erosion process was substantially accelerated because a large amount of water was released downstream. The breach simultaneously developed in both vertical and lateral directions. The outflow discharge rapidly increased (*r*_2_ = 0.120, and *r*_2_ ≫ *r*_1_) and reached the peak (*Q_p_* = 2.86 L/s), seen in Figure 10. Stage II ended when the apparent erosion stopped at the breach entrance. During Stage III (the attenuating and reequilibrium stage), the erosion balance was achieved, and the breach reached its final dimensions. The outflow discharge gradually attenuated and eventually equalized with the inflow discharge. The whole breaching process of landslide dams ended.

The breaching duration of landslide dams differed depending on the dam material and inflow discharge, as shown in Table 3. With the same inflow discharge, the breaching duration *T* for the fine-grained dam was longer than that of the well-graded dam. More specifically, the duration of Stage I *t_I_* for the fine-grained dam was much shorter than the value of the well-graded dam, but the durations of Stages II *t_II_* and III *t_III_* for the fine-grained dam were longer than the values of the well-graded dam, respectively. With the same dam material, the breaching duration *T* decreased with the increasing of inflow discharge *Q_in_*. More specifically, the inflow discharge changed the breaching duration for the fine-grained dam mainly by affecting the breach development stage (Stage II) and for the well-graded dam mainly by affecting the breach initiation stage (Stage I). Under the extreme inflow condition (*Q_in_* = 2.0 L/s), the coarse-grained dam failed by overtopping in a short duration (*T* = 95 s), which meant the pressing early warning time for the dam breaching.

### 3.3. Breach Development and Erosion Rates

**Figure 11:**
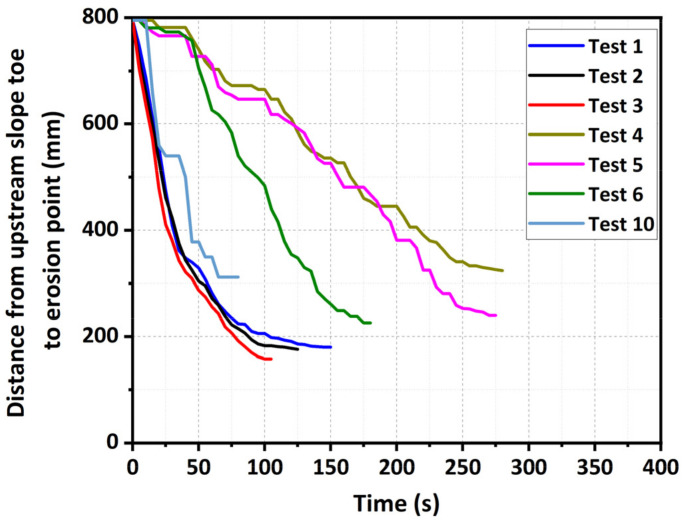
Movement process of erosion rates.

The erosion rates in different directions of landslide dams were summarized in Table 4. The headward erosion significantly developed during the breaching process. The movement processes of the erosion points in the experiments were shown in Figure 11. The erosion point was the same as the invert point on the dam longitudinal profile, which indicated the loss of control and the retreat of breach channel [60]. The experimental results revealed that, with the same inflow discharge, the headward erosion rate *E_h_* for the fine-grained dam was larger than the value for the well-graded dam (Table 4), because the shear strength of the fine-grained material was lower. With the same dam material, the headward erosion rate *E_h_* for the fine-grained dam linearly increased with an increase in the inflow discharge *Q_in_* (Tests 1–3). However, the headward erosion rate *E_h_* for the well-graded dam nonlinearly increased (Tests 4–6): its increasing value was amplified by the increasing inflow discharge (Table 4). The reason for this discrepancy was that the headward erosion rate was simultaneously associated to the flow velocity and scarp height [61,62]. The larger inflow discharge significantly increased the scarp height for the well-graded dams (Figure 9), inducing more potential energy of the stored water to be transformed into the kinetic energy. For the coarse-grained dam when *Q_in_* = 2.0 L/s (Test 10), the erosion point intermittently moved towards the upstream slope toe but at a high average speed (*E_h_* = 6.19 mm/s), as shown in Figure 11. This was because of the dam body slide and the grain transportation process similar to a debris flow, which almost instantaneously improved the headward speed of the erosion point.

The breach was deepened by the vertical erosion and broadened by the lateral erosion during the breaching process. The experimental results revealed that, with the same inflow discharge, both the vertical and lateral erosion rates *E_v_*, *E_l_* decreased with an increase in the median diameter *d*_50_ of materials (Table 4) because the finer grains were easier to wash away than the coarser grains [63,64]. The fine content *p* of materials affected the erosion rates by changing the median diameter and void ratio. The median diameter increased when the fine content decreased, and the coarse content increased (Table 2). With the same dam material, both the vertical and lateral erosion rates *E_v_*, *E_l_* increased with an increase in the inflow discharge *Q_in_* because the increasing inflow discharge enhanced the erosion ability of the outflow.

**Figure 12:**
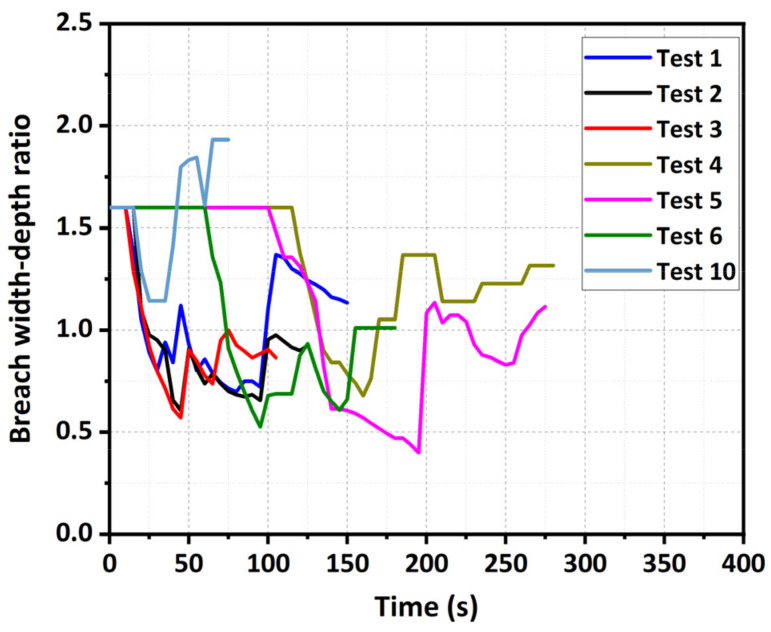
Relationship curve between time and breach width–depth ratio.

The development of the ratio of breach top width to breach depth *B_t_*/*D* in the experiments was shown in Figure 12. The experimental results revealed that all the changing curves of the breach width–depth ratio *B_t_*/*D* were nonlinear with time. More specifically, the vertical erosion was the predominant erosion mechanism during Stage I, and then the vertical and lateral erosions simultaneously developed during Stage II. Finally, the lateral erosion became predominant in the early phase of Stage II. With the same inflow discharge, the final breach width–depth ratio *B_t_*/*D* for the fine-grained dam was smaller than the value for the well-graded dam (Figure 12). This breach development result was consistent with the observations of Jiang et al. (2018) [38]. With the same dam material, the final breach width-depth ratio *B_t_*/*D* decreased with an increase in the inflow discharge *Q_in_* (Figure 12). The relationships between the final breach width–depth ratio, fine content, and inflow discharge reflected that the increasing fine content and inflow discharge were more inclined to strengthen the vertical erosion rate than the lateral erosion rate. The reason was that the breaching duration obviously decreased with an increase in the fine content and inflow discharge (Table 3), inducing a rapid decrease in the upstream water level, and the vertical erosion on the breach channel developed earlier than the lateral erosion. Consequently, the final breach of the dam with more fine content was deeper and narrower than that of the dam with less fine content. For the coarse-grained dam when *Q_in_* = 2.0 L/s (Test 10), it was interesting that the final breach width–depth ratio (*B_t_*/*D* = 1.93) was larger than the initial value (Figure 12). One reason was that the erosional resistance of the coarse-grained material was so large that the breach depth was relatively small (Table 4). On the other hand, the loss of coarse grains during dam body slides substantially loosened the dam structure, leading to the frequent occurrences of lateral collapses with larger volumes.

### 3.4. Hydrological Evolution

**Figure 13:**
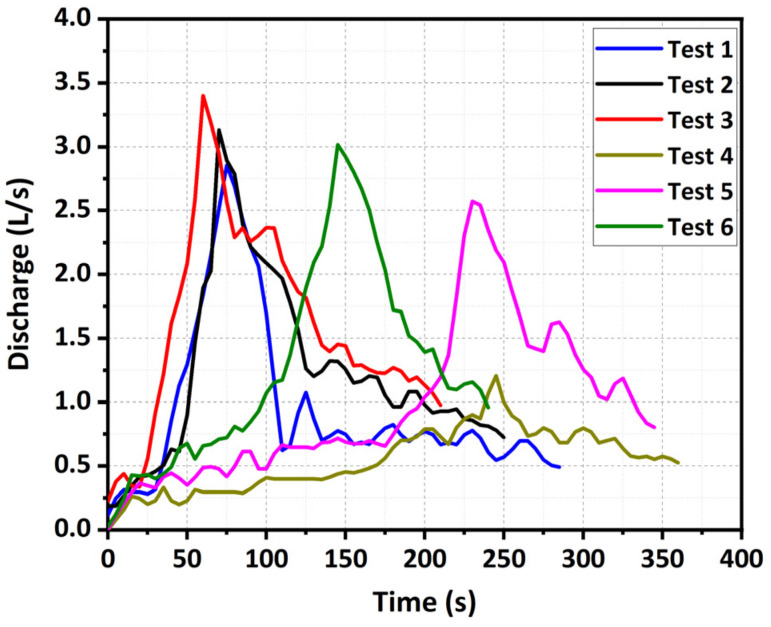
Outflow discharge hydrographs of Tests 1–6.

The outflow discharge hydrographs for Tests 1–6 in the experiments were shown in Figure 13. The experimental results revealed that the dam material and inflow discharge had substantial effects on the hydrological shape of the outflow. With the same inflow discharge, the hydrograph shape tended to vary from a lanky one to a broad one as the fine content *p* decreased (Figure 13). Compared with the well-graded dam, the fine-grained dam had a larger peak outflow discharge *Q_p_* and an earlier arrival time of the peak *t_p_*, as shown in Table 3. This hydrological result was consistent with the observations of Coleman et al. (2002) [9]. Because of this, the smaller mean diameter of materials led to the smaller erosional resistance and larger erosion rates (Table 4), and the outflow discharge *Q_out_* could be calculated using the broad-crest weir equation [65,66]:

(2) $$Q_{out}=CB_{w}{\left(H-Z\right)}^{3/2}$$

where *C* is a discharge coefficient, *B_w_* is the cross-section width, *H* is the reservoir height, and *Z* is the elevation of the breach bottom. Based on the equation, the larger erosion rate could lead the relative height between the upstream water level and the breach bottom to increase, which increased the outflow discharge during the breaching process.

**Figure 14:**
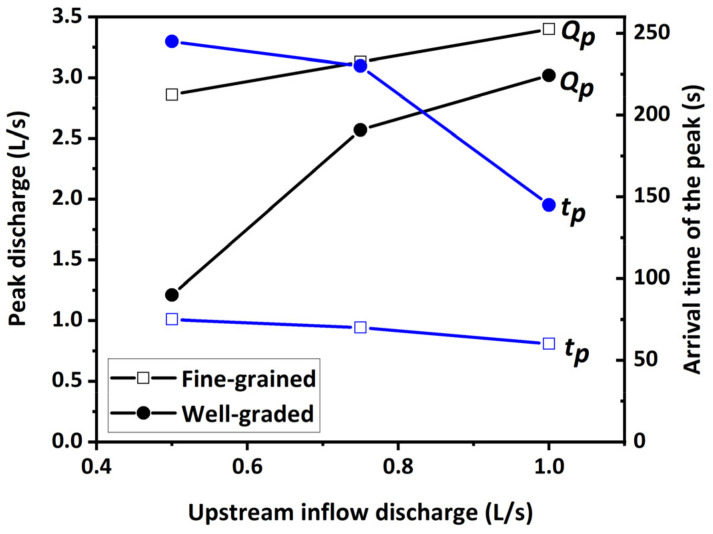
Influence of material and inflow discharge on the peak outflow discharges.

**Figure 15:**
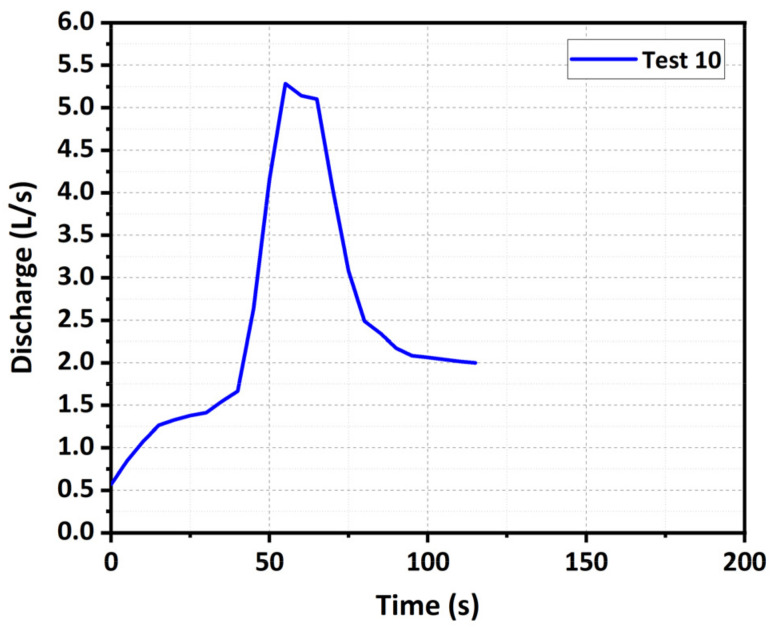
Outflow discharge hydrograph of Tests 10.

With the same dam material, the larger the inflow discharge *Q_in_* was, the larger the peak outflow discharge *Q_p_* was, and the earlier the arrival time of the peak *t_p_* was, as shown in Table 3 and Figure 14. The reason was that the larger inflow discharge caused a higher upstream water level at the end of Stage I, which led to a stronger erosion force acting on the breach channel. Nevertheless, the linear relationship between the arrival time of the peak and inflow discharge for the fine-grained dams (Tests 1–3) was very weak (Figure 14) because the duration of Stage I hardly changed with an increase in the inflow discharge (Table 3). Unlike the fine-grained dams, the duration of Stage I for the well-graded dams obviously decreased with an increase in the inflow discharge (Table 3), which significantly shortened the arrival time of the peak. This above discrepancy was related to the distinct breaching characteristics, which basically resulted from the water–sediment interactions during the breaching process. The experimental results also revealed that the disparity of the peak discharge between the fine-grained dam and well-graded dam was gradually reduced with an increase in the inflow discharge *Q_in_* (Figure 14). The reason was that, compared with the fine-grained dam, the erosion rates in the vertical and lateral directions for the well-graded dam increased faster with an increase in the inflow discharge (Table 4), accelerating the increase of the outflow discharge. Moreover, the hydrograph shape tended to vary from a multimodal one to a unimodal one with an increase in the inflow discharge *Q_in_*. Because of this, when the inflow discharge was smaller (i.e., *Q_in_* = 0.5 L/s), the sediments from lateral collapses might have blocked the breach channel temporarily and interrupted the outflow, resulting in a peak in the hydrograph. As the blockage was scattered and pushed away, the outflow continued and reached another peak discharge. However, when the inflow discharge was larger (i.e., *Q_in_* = 1.0 L/s), the outflow was large enough to push away the sediments in the breach quickly. For the coarse-grained dam, when *Q_in_* = 2.0 L/s (Test 10), the discharge hydrograph was shaped into a very thin, high type (Figure 15), because the upstream water level increased first and then decreased rapidly as the dam failed by overtopping in a short time. The extreme large inflow discharge considerably overwhelmed the effects of dam material on the outflow discharge and tremendously increased the risks and dangers of the dam breaching.

## 4. Discussion

### 4.1. Erosion Rate Distribution along the Flow Direction

**Figure 16:**
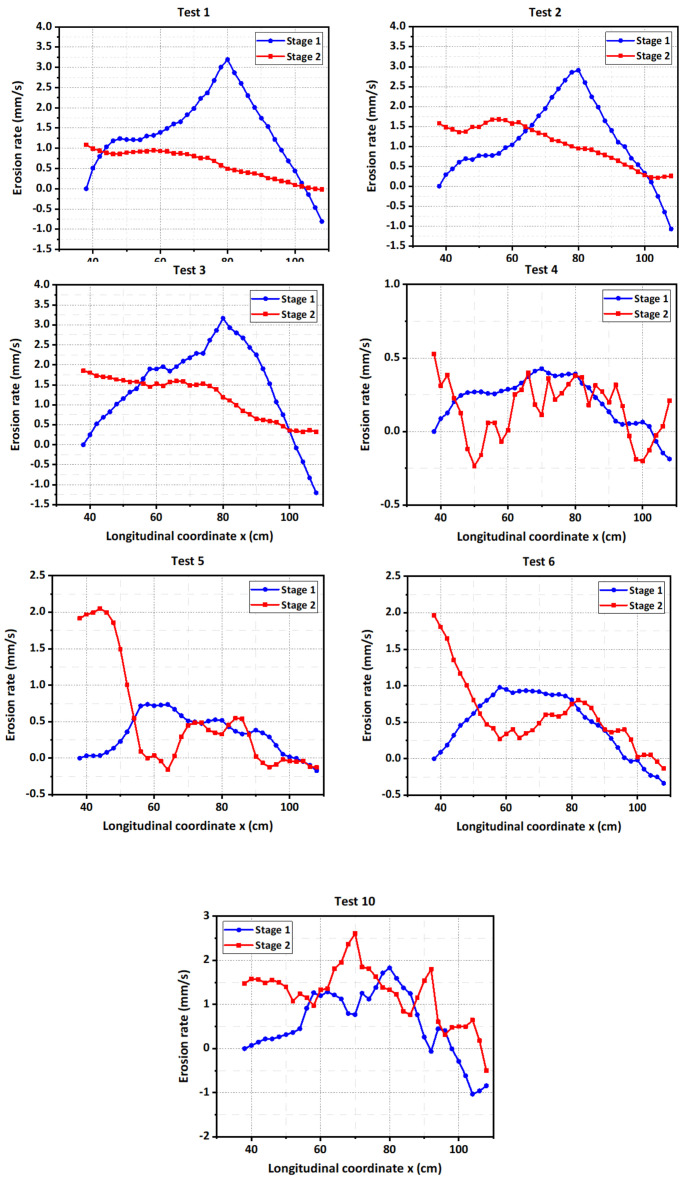
Distribution of erosion rate in two stages for the experimental tests.

Based on the changes in the elevation of the breach bottom at some time intervals, the rate of the erosion *E_i_* which imposed on the breach channel during the breaching process could be calculated:

(3) $$E_{i}=\frac{\Delta d_{i}}{\Delta t_{i}}$$

where *E_i_* is the erosion rate, and $\Delta d_{i}$ is the erosion depth during the time interval $\Delta t_{i}$. The distributions of the erosion rates along the flow direction during Stages I and II in the experiments were shown in Figure 16.

For the fine-grained dams, the erosion rate in Stage I first increased and then decreased along the flow direction, and the erosion rate in Stage II overall decreased (Figure 16). The movement process of the location of the maximum erosion rate was fairly consistent for all inflow discharges (*Q_in_* = 0.5, 0.75 and 1.0 L/s). More specifically, the maximum point moved away from near the downstream crest in Stage I, and then reached the upstream slope in Stage II (Figure 16). The reason was that the breach channel approximately retreated in parallel during the breaching process, and most times, the downstream slope sustained a relative constant angle. The experimental results also revealed that the maximum erosion rate in Stage I was larger than the maximum value in Stage II because the fine grains were still easy to wash away under the condition of the small flow velocity, and the duration of Stage I was much smaller than the value of Stage II. For the same reasons, the increasing inflow discharge slightly increased the maximum erosion rate in Stage I but relatively significantly enhanced the maximum erosion rate in Stage II (Figure 16).

For the well-graded dams, the distribution of the erosion rates along the flow direction were different from those of the fine-grained dams and became more complicated (Figure 16). The erosion rate in Stage I first increased and then decreased along the flow direction similar to the fine-grained dams, but the changing curve was relatively unsmooth. The main difference was that there were many fluctuations in the changing curve of the erosion rates in Stage II (Figure 16). The primary reason for this discrepancy was that, for the well-graded dams, a step-pool or an approximately step-pool structure was generated on the dam longitudinal profile due to the scarp scour, resulting in an undulating surface of dam during the breaching process. Especially in Test 4 (*Q_in_* = 0.5 L/s), as the inflow discharge was too small to transport coarse grains at some locations, the unstable coarse grains were transported downstream very slowly and even deposited again on the breach channel. This process developed simultaneously with the scarp migration, causing the changing curve of the erosion rates for Test 4 to fluctuate most violently (Figure 16). In Tests 5 (*Q_in_* = 0.75 L/s) and 6 (*Q_in_* = 1.0 L/s), the maximum erosion rate points were near the middle of the dam crest in Stage I, and then were located on the upstream slope in Stage II (Figure 16). The reason was that, combining the evolutions of the dam longitudinal section (Figure 9), the larger inflow discharge induced a larger and deeper scarp in the middle of the dam crest in Stage I, and then, the outflow with a strong erosional force severely eroded the upstream slope and filled the previous scarp in Stage II. Thus, an obvious inflection point in the changing curve of the erosion rates in Stage II—a sudden negative change at the location of the scarp—could be observed (Figure 16). Different from the fine-grained dams, the increasing inflow discharge simultaneously enhanced the maximum erosion rates for the well-graded dams in Stage I and II, which also explained the discrepancy in the movement process of the maximum point between Test 4 and Tests 5–6.

For the coarse-grained dam when *Q_in_* = 2.0 L/s (Test 10), the overall distribution rule of the erosion rates along the flow direction in Stage I was basically consistent in the rule for other tests (first increasing and then decreasing), as shown in Figure 16. However, the changing curve of the erosion rates in Stage II fluctuated up and down (Figure 16). In addition, the maximum erosion rate point was always located near the downstream crest during the breaching process. The reason for the interesting results was that most coarse grains could not be immediately washed away and entrained into the downstream like the fine grains but were transported step by step on the breach channel. The above results further described the water–sediment interactions during the breaching process of landslide dams, and they also reflected that the extreme large inflow discharge could partially but not completely overwhelm the effects of coarse-grained material on the erosion characteristics.

The experimental results indicated that the distribution of erosion rates was significantly affected by the dam material and inflow discharge. The erosion rate may vary in a nonlinear way along the flow direction. However, the distributions of erosion rates in existing longitudinal evolution models of landslide dams are inconsistent with these observed results, which may lead to differences in the erosion process from the actual event. For example, Chang and Zhang (2010) assumed that the erosion rate on the downstream slope remained the same at each time step [33]. Zhong et al. (2018) assumed that the erosion rate decreased along the flow direction [34]. Therefore, the distribution of erosion rates along the flow direction should be simulated in stages based on the dam material type and inflow condition in the future breach models.

### 4.2. Erosion Resistance of Dam Materials

The erodibility of materials was one of the most important intrinsic factors governing the erosion process of landslide dams that failed by overtopping. The breach erosion process was determined by the interactions between the water flow and soil. A classical shear stress equation [67] was often used to describe the soil erosion:

(4) $$E=K_{d}\left(\tau -\tau_{c}\right)$$

where *E* is the erosion rate of soil (mm/s); *τ* is the bed shear stress (Pa); *K_d_* is the coefficient of erodibility (mm^3^/N-s), and *τ_c_* is the critical shear stress (Pa). The erosion resistance of soil was represented by *K_d_* and *τ_c_*, in which *τ_c_* reflected the threshold for the erosion initiation, and *K_d_* reflected how fast the soil could be eroded. The shear stress *τ* induced by the water flow reflected the violence of the flow conditions within the breach channel. To ease calculations, the outburst floods during the breaching process of landslide dams was assumed to be uniform flow [68,69,70]. The shear stress acting on the sloping bed was often estimated based on Manning’s equation [34,56,71]:

(5) $$\tau =\frac{\rho_{w}gn^{2}v^{2}}{R^{1/3}}$$

where *ρ*_w_ is the water density (1000 kg/m^3^); *g* is the acceleration due to gravity; *n* is the Manning coefficient; *v* is the velocity of fluid, and *R* is the hydraulic radius. The value of the Manning coefficient *n* is related to the bedload median diameter *d*_50_ (in meter):

(6) $$n=\frac{d_{50}^{1/6}}{A_{n}}$$

where *A_n_* is an empirical coefficient, and *A_n_* = 16 for the laboratory scale [72].

**Figure 17:**
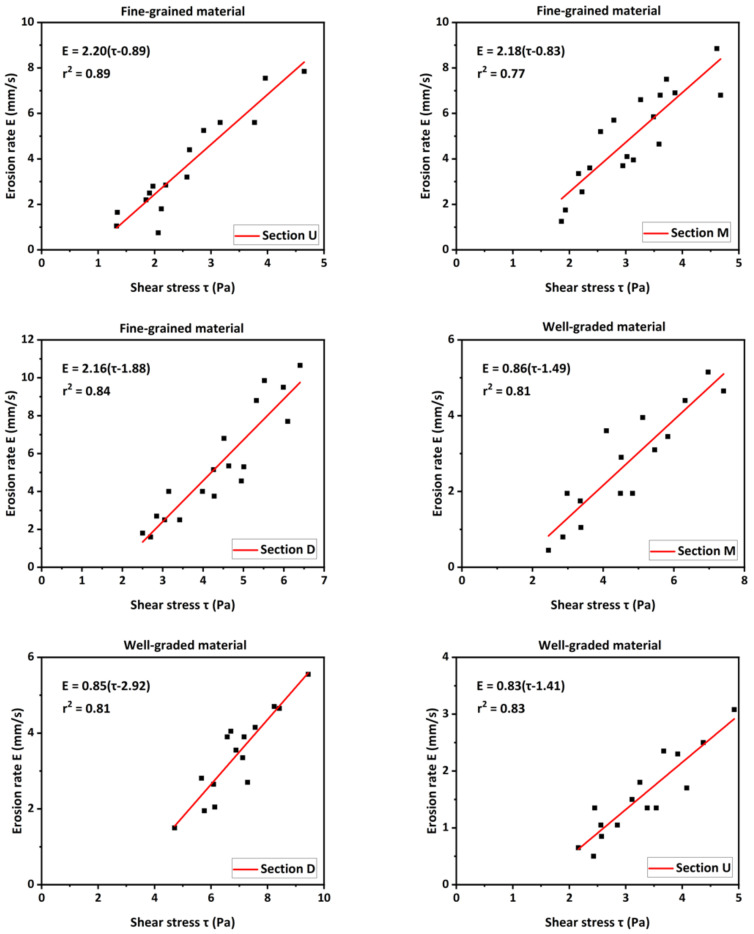
Relationship between erosion rate and shear stress at three cross-sections.

To find the relationship between the erosion rate and shear stress, three certain cross-sections of dam, including the upstream crest (Section ‘U’), the middle of the dam crest (Section ‘M’), and the downstream crest (Section ‘D’), were chosen for measuring. The erosion rate *E* and shear stress *τ* at the cross-sections ‘U’, ‘M’, and ‘D’ were calculated in the experiments, respectively. The values were plotted against each other to quantify the erosion resistance of dam materials, as shown in Figure 17. The experimental results further verified the linear relationship between the erosion rate and shear stress. Nevertheless, it was noteworthy that this linear relationship was not applicable at Sections ‘U’ and ‘M’ for the early phase of Stage I and at Section ‘D’ for last phase of Stage II. The reason might be that the outflow discharge was so small that the flow velocity relatively fluctuated in a short time in the early phase of Stage I, and the inclination angle of the breach channel was too small in the last phase of Stage II.

The coefficient of erodibility *K_d_* and the critical shear stress *τ_c_* of materials at different dam cross-sections could be quantified by linear fitting of these points which represented the erosion rate and shear stress (Figure 17). The values of *K_d_* and *τ_c_* of materials in the experiments were summarized in Table 5. The experimental results revealed that, with the same dam material, as these lines were approximately parallel (Figure 17), the coefficient of erodibility *K_d_* was almost the same at dam different cross-sections (Table 5). The relative invariance of *K_d_* throughout the dam body was because of the well-controlled conditions when the dam was constructed [70]. However, the critical shear stress *τ_c_* obviously changed at different dam cross-sections (Figure 17). More specifically, the critical shear stress *τ_c_* at Section ‘D’ was larger than the values at Sections ‘U’ and ‘M’ (Table 5). This above result was approximately consistent with the findings of Zhou et al. (2019) [19]. One possible reason for the variation in the critical shear stress along the flow direction was that the capacity of the water flow to entrain sediments decreased as the sediment concentration *C*_s_ increased from upstream to downstream [73].

With the same inflow discharge, the value of *τ_c_* increased and *K_d_* decreased with an increase in the median diameter *d*_50_ of materials (Table 5). The experimental results revealed that the finer the grains were, the easier they eroded and the faster the erosion rate was. This could reasonably explain the difference of breaching process and breach development for dams with different grain compositions. Furthermore, quantitative comparisons of the erosion resistance based on the experimental results and calculated results from empirical equations [33,74,75] were summarized in Table 5. The experimental results revealed that the erosion resistance substantially depended on the grain size distribution and soil parameters, and the experimental results at some specific cross-sections of dam were close to the values calculated from several empirical equations. Nevertheless, if the above interpretation of the variation in the critical shear stress along the flow direction was correct, the variation seemed not to be fully considered in existing empirical equations, which might cause the erosion process to go beyond the expectation range.

### 4.3. Effects of Dam Material on the Lateral Collapse

The breach was broadened due to lateral collapses during the breaching process. The statistics of the occurrence of lateral collapses in the experiments were shown in Table 6. The experimental results revealed that the lateral collapse was significantly affected by the dam material but not inflow discharge.

Compared with the fine-grained dams, the total number of lateral collapses for the well-graded dams increased by approximately 124% to 213% (Table 6). One reason was that, with the same inflow discharge, the breaching duration for the well-graded dam was considerably longer than the value for the fine-grained dam (Table 3). On the other hand, the vertical erosion rate for the well-graded dam was smaller, causing more lateral collapses to occur in the process of the breach’s slow deepening (Table 4). Almost all lateral collapses for the fine-grained dams occurred in the breach development stage (Stage II), but more than half for the well-graded dams occurred in the breach initiation stage (Stage I) because the duration of Stage I for the fine-grained dam was much shorter (Table 3). The experimental results also revealed that 53–65% of lateral collapses for the fine-grained dams occurred on the downstream slope, and 55–57% of them for the well-graded dams occurred in the middle of the dam. The reason for this discrepancy was that the downstream slope was almost flattened for the fine-grained dam, but the breach in the middle of the dam was deepened most obviously for the well-graded dam (Figure 16). Moreover, the average volume of lateral collapses for the fine-grained dams was larger than the value for the well-graded dams because the breach for the fine-grained dam was deeper with a larger vertical erosion rate (Table 4). Overall, the influence of dam material on the spatial and temporal distribution of lateral collapses is seldom considered in existing physically based breach models [32,33]. The occurrence and distribution of lateral collapses vary with the type of dam material, which should be further studied in the future models to improve the simulation accuracy of breach lateral enlargement.

## 5. Conclusions

Based on a series of physical flume tests, the breaching process of landslide dams with differing grain compositions under different inflow conditions were investigated and compared in this study. Experimental results revealed that the breaching process of landslide dams was determined by the water-sediment interaction. The failure mode of dams varied with dam material and inflow discharge, which means the dam materials and inflow conditions should be considered simultaneously in practical engineering. Through the experimental results, three stages of the breaching process were generalized. The final breach of the dam with higher fine content was deeper and narrower. The positive relationship between the peak discharge and inflow discharge was further identified. In addition, there were many fluctuations in the changing curve of erosion rates along the flow direction in Stage II for the well-graded as well as the coarse-grained dams, which were different from the hydrological processes in existing longitudinal evolution models of landslide dams. Moreover, the erosion resistance of dam materials increased along the flow direction. The relationship between the erosion rate and the shear stress at different cross-sections of the dam needs to be further considered in physically based breach models to reduce inaccuracies. Finally, the lateral collapse, which was usually neglected in existing studies, was affected by the dam material instead of inflow discharge.

The breaching process of landslide dams is a complicated process involving geotechnical and hydro-dynamic mechanisms. More factors should be studied in future studies such as monitoring the flow velocity and internal erosion, considering the effects of scaling and real-world factors in more reasonable ways, and considering the inhomogeneities in landslide dam structure.

## Figures and Tables

**Table 1:** Characteristic dimensions of model dam and natural landslide dams.

	*H_d_*/*C_d_*	*S_u_*	*S_d_*	$H_{d}/B_{d}$	$V_{d}^{1/3}/H_{d}$	$V_{l}^{1/3}/H_{d}$
Model dam	1	26.6°	33.7°	0.22	1.66	2.53
Natural landslide dams	0.2–3.0	11–45°	11–45°	0.02–1	0.5–5	0.2–10

Note: Dam height = *H_d_*; Dam crest with = *C_d_*; Upstream slope = *S_u_*; Downstream slope = *S_d_*; Dam bottom width = *B_d_*; Dam volume = *V_d_*; Barrier lake volume = *V_l_*.

**Table 2:** Summary of the experimental tests.

Test No.	Dam Material	*ρ_d_* (kg/m^3^)	*d*_50_ (mm)	*p* (%)	*Q_in_* (L/s)
1	Fine-grained	1780	0.8	50.2	0.5
2					0.75
3					1.0
4	Well-graded	1780	3.8	33.5	0.5
5					0.75
6					1.0
7	Coarse-grained	1780	13.6	10.3	0.5
8					0.75
9					1.0
10					2.0

Note: dry density = *ρ_d_*; median diameter = *d*_50_; fine content = *p* (the fines in dam materials are grains whose diameters are less than 0.075 mm); inflow discharge = *Q_in_*.

**Table 3:** Breaching durations and discharges of the experimental tests.

Test No.	Failure Mode	Stage I			Stage II				Stage III	*T* (s)
		*t*_1_ (s)	*Q*_1_ (L/s)	*r*_1_	*t*_2_ (s)	*Q*_p_ (L/s)	*t*_p_ (s)	*r*_2_	*t*_3_ (s)	
1	O	32	0.29	0.018	116	2.86	75	0.120	139	287
2	O	34	0.55	0.022	87	3.13	70	0.096	128	249
3	O	31	0.80	0.026	73	3.40	60	0.090	107	211
4	O	221	0.70	0.006	58	1.21	245	0.043	83	362
5	O	197	0.97	0.007	76	2.57	230	0.065	72	345
6	O	114	1.21	0.011	64	3.02	145	0.058	63	241
7	N	/								
8	N	/								
9	S/N	/								
10	O	41	1.79	0.022	37	5.29	55	0.125	17	95

Note: overtopping failure = O; no failure (stable status) = N; shallow slide = S; duration of Stage II = *t*_2_; duration of Stage III = *t*_3_; breaching duration (*t*_1_ + *t*_2_ + *t*_3_) = *T*.

**Table 4:** Erosion rates of the experimental tests.

Test No.	*t*_1–2_ (s)	Headward Erosion			Vertical Erosion			Lateral Erosion		
		*d* (mm)	Δd (mm)	*E_h_* (mm/s)	*Z* (mm)	ΔZ(mm)	*E_v_* (mm/s)	*B_t_* (mm)	$\Delta B_{t}\;(\mathrm{mm})$	*E_l_* (mm/s)
1	148	180	615	4.16	37	153	1.03	230	150	1.01
2	121	176	619	5.12	18	172	1.42	205	125	1.03
3	104	158	637	6.13	20	170	1.63	190	110	1.06
4	279	324	471	1.69	126	64	0.23	150	70	0.25
5	273	240	555	2.03	65	125	0.46	195	115	0.42
6	178	226	569	3.20	60	130	0.73	182	102	0.57
7–9	No failure									
10	78	312	483	6.19	91	99	1.27	288	208	2.67

Note: duration of Stage I and II (*t*_1_ + *t*_2_): *t*_1–2_; distance from upstream slope toe to erosion point: *d*; value between the initial and final distance from upstream slope toe to erosion point: ∆*d*; average rate of headward erosion: *E_h_*; breach bottom elevation: Z; value between the initial and final breach bottom elevation: ∆Z; average rate of vertical erosion: *E_v_*; breach top width: *B_t_*; value between the initial and final breach top width: ∆*B_t_*; average rate of lateral erosion: *E_l_*.

**Table 5:** Coefficient of erodibility and critical shear resistance of materials.

Dam Material	The Coefficient of Erodibility *K_d_* (m^3^/N-s)			The Critical Shear Stress *τ_c_* (Pa)		
Fine-grained	Experimental results					
	‘U’	‘M’	‘D’	‘U’	‘M’	‘D’
	2.20	2.18	2.16	0.89	0.83	1.88
	Calculated results from empirical equations					
	2.20			1.97		
				0.69		
Well-graded	Experimental results					
	‘U’	‘M’	‘D’	‘U’	‘M’	‘D’
	0.83	0.86	0.85	1.41	1.49	2.92
	Calculated results from empirical equations					
	1.03			3.14		
				1.22		

**Table 6:** Distribution of lateral collapses for the experimental tests.

Test No.	Total Number	Spatial Distribution			Time Distribution	
		Upstream Slope	Dam Middle	Downstream Slope	Stage I	Stage II
1	15	2	5	8	3	12
2	17	1	5	11	3	14
3	16	0	6	10	3	13
4	47	1	27	19	27	20
5	38	2	21	15	25	13
6	45	4	25	16	31	14

## Data Availability

Data sharing not applicable.

## References

[1] Costa J.E., Schuster R.L. (1991). Documented historical landslide dams from around the world. Open-File Rep..

[2] Casagli N., Ermini L., Rosati G. (2003). Determining grain size distribution of the material composing landslide dams in the Northern Apennines: Sampling and processing methods. Eng. Geol..

[3] Shen D., Shi Z., Peng M., Zhang L., Jiang M. (2020). Longevity analysis of landslide dams. Landslides.

[4] Peng M., Jiang Q.-L., Zhang Q.-Z., Hong Y., Jiang M.-Z., Shi Z.-M., Zhang L.-M. (2019). Stability analysis of landslide dams under surge action based on large-scale flume experiments. Eng. Geol..

[5] Chang L.C. (1938). Investigation of Diexi Earthquake in Sichuan Province. Geol. Rev..

[6] Liu N., Cheng Z.L., Cui P., Chen N.S. (2013). Dammed Lake and Risk Management.

[7] Hu X.W., Huang R.Q., Shi Y.B., Lu X.P., Zhu H.Y., Wang X.R. (2009). Analysis of blocking river mechanism of Tangjiashan landslide and dam-breaking mode of its barrier dam. Chin. J. Rock Mech. Eng..

[8] Fujita Y., Tamura T. (1987). Enlargement of breaches in flood levees on alluvial plains. Nat. Disaster Sci..

[9] Coleman S.E., Andrews D.P., Webby M.G. (2002). Overtopping Breaching of Noncohesive Homogeneous Embankments. J. Hydraul. Eng..

[10] Spinewine B., Delobbe A., Elslander L., Zech Y. (2004). Experimental investigation of the breach growth process in sand dikes. Proceedings of the River Flow 2004.

[11] Braun A., Cuomo S., Petrosino S., Wang X., Zhang L. (2017). Numerical SPH analysis of debris flow run-out and related river damming scenarios for a local case study in SW China. Landslides.

[12] Peng M., Zhang L.M. (2011). Breaching parameters of landslide dams. Landslides.

[13] Ermini L., Casagli N. (2003). Prediction of the behaviour of landslide dams using a geomorphological dimensionless index. Earth Surf. Process. Landf..

[14] Xu F.-G., Yang X.-G., Zhou J.-W., Hao M.-H. (2013). Experimental Research on the Dam-Break Mechanisms of the Jiadanwan Landslide Dam Triggered by the Wenchuan Earthquake in China. Sci. World J..

[15] Schmocker L., Frank P.-J., Hager W.H. (2014). Overtopping dike-breach: Effect of grain size distribution. J. Hydraul. Res..

[16] Yang Y., Cao S.Y., Yang K.J., Li W.P. (2015). Experimental study of breach process of landslide dams by overtopping and its initiation mechanisms. J. Hydrodyn..

[17] Zhao W., Chen X., You Y., Chen J. (2015). Dam-break characteristics of landslide dams with different types of open channel discharge sections. Environ. Earth Sci..

[18] Zhao T.-L., Chen S.-S., Fu C.-J., Zhong Q.-M. (2019). Centrifugal model tests and numerical simulations for barrier dam break due to overtopping. J. Mt. Sci..

[19] Zhou G.G., Zhou M., Shrestha M.S., Song D., Choi C., Cui K.F.E., Peng M., Shi Z., Zhu X., Chen H. (2019). Experimental investigation on the longitudinal evolution of landslide dam breaching and outburst floods. Geomorphology.

[20] Schuster R.L., Costa J.E. (1986). A Perspective on Landslide Dams. Landslide Dams, Processes, Risk and Mitigation. https://pubs.er.usgs.gov/publication/70015581.

[21] Chang D.S., Zhang L.M., Xu Y., Huang R.Q. (2011). Field testing of erodibility of two landslide dams triggered by the 12 May Wenchuan earthquake. Landslides.

[22] Schuster R.L. Landslide dams in the western United State. Proceedings of the IVth International Conference and Field Workshop on Landslides.

[23] Korup O. (2004). Geomorphometric characteristics of New Zealand landslide dams. Eng. Geol..

[24] Cui P., Zhu Y.Y., Han Y.S., Chen X.Q., Zhuang J.Q. (2009). The 12 May Wenchuan earthquake-induced landslide lakes: Dis-tribution and preliminary risk evaluation. Landslides.

[25] Gorum T., Fan X., van Westen C.J., Huang R.Q., Xu Q., Tang C., Wang G. (2011). Distribution pattern of earthquake-induced landslides triggered by the 12 May 2008 Wenchuan earthquake. Geomorphology.

[26] Hou J., Liang Q., Simons F., Hinkelmann R. (2013). A 2D well-balanced shallow flow model for unstructured grids with novel slope source term treatment. Adv. Water Resour..

[27] Pu J.H., Shao S., Huang Y., Hussain K. (2013). Evaluations of SWEs and SPH Numerical Modelling Techniques for Dam Break Flows. Eng. Appl. Comput. Fluid Mech..

[28] Pu J.H., Hussain K., Shao S.-D., Huang Y.-F. (2014). Shallow sediment transport flow computation using time-varying sediment adaptation length. Int. J. Sediment Res..

[29] Singh V.P., Quiroga C.A. (1987). A dam-breach erosion model: I. Formulation. Water Resour. Manag..

[30] Fread D.L. (1988). BREACH: An Erosion Model for Earth Dam Failures.

[31] Mohamed M.A.A. (2002). Embankment Breach Formation and Modeling Methods. Ph.D Thesis.

[32] Wang L., Chen Z., Wang N., Sun P., Yu S., Li S., Du X. (2016). Modeling lateral enlargement in dam breaches using slope stability analysis based on circular slip mode. Eng. Geol..

[33] Chang D.S., Zhang L. (2010). Simulation of the erosion process of landslide dams due to overtopping considering variations in soil erodibility along depth. Nat. Hazards Earth Syst. Sci..

[34] Zhong Q.M., Chen S.S., Mei S.A., Cao W. (2017). Numerical simulation of landslide dam breaching due to overtopping. Landslides.

[35] Gregoretti C., Maltauro A., Lanzoni S. (2010). Laboratory Experiments on the Failure of Coarse Homogeneous Sediment Natural Dams on a Sloping Bed. J. Hydraul. Eng..

[36] Cao Z., Yue Z., Pender G. (2011). Landslide dam failure and flood hydraulics. Part I: Experimental investigation. Nat. Hazards.

[37] Chen S.-C., Lin T.-W., Chen C.-Y. (2015). Modeling of natural dam failure modes and downstream riverbed morphological changes with different dam materials in a flume test. Eng. Geol..

[38] Jiang X.G., Huang J.H., Wei Y.W., Niu Z.P., Chen F.H., Zou Z.Y., Zhu Z.Y. (2018). The influence of materials on the breaching process of natural dams. Landslides.

[39] Zhu X., Peng J., Liu B., Jiang C., Guo J. (2020). Influence of textural properties on the failure mode and process of landslide dams. Eng. Geol..

[40] Peng M., Zhang L., Chang D., Shi Z. (2014). Engineering risk mitigation measures for the landslide dams induced by the 2008 Wenchuan earthquake. Eng. Geol..

[41] Shi Z.M., Zhang G.D., Peng M., Ma C.Y. (2021). Influence of dam geometry on the breaching process of landslide dams. Proceedings of the IOP Conference Series: Earth and Environmental Science.

[42] Zheng H., Shi Z., Shen D., Peng M., Hanley K.J., Ma C., Zhang L. (2021). Recent Advances in Stability and Failure Mechanisms of Landslide Dams. Front. Earth Sci..

[43] Pu J. (2021). Velocity Profile and Turbulence Structure Measurement Corrections for Sediment Transport-Induced Water-Worked Bed. Fluids.

[44] Pu J.H. (2018). Turbulent rectangular compound open channel flow study using multi-zonal approach. Environ. Fluid Mech..

[45] Pu J.H., Wei J., Huang Y. (2017). Velocity Distribution and 3D Turbulence Characteristic Analysis for Flow over Water-Worked Rough Bed. Water.

[46] Zheng H., Shi Z., Yu S., Fan X., Hanley K.J., Feng S. (2021). Erosion Mechanisms of Debris Flow on the Sediment Bed. Water Resour. Res..

[47] Peng M., Ma C.-Y., Chen H.-X., Zhang P., Zhang L.-M., Jiang M.-Z., Zhang Q.-Z., Shi Z.-M. (2021). Experimental study on breaching mechanisms of landslide dams composed of different materials under surge waves. Eng. Geol..

[48] Shi Z.-M., Wang Y.-Q., Peng M., Chen J.-F., Yuan J. (2015). Characteristics of the landslide dams induced by the 2008 Wenchuan earthquake and dynamic behavior analysis using large-scale shaking table tests. Eng. Geol..

[49] Yin Y., Wang F., Sun P. (2009). Landslide hazards triggered by the 2008 Wenchuan earthquake, Sichuan, China. Landslides.

[50] Beyabanaki S.A.R., Bagtzoglou A.C., Liu L. (2015). Applying disk-based discontinuous deformation analysis (DDA) to simulate Donghekou landslide triggered by the Wenchuan earthquake. Géoméch. Geoengin..

[51] Li X., He S., Luo Y., Wu Y. (2011). Simulation of the sliding process of Donghekou landslide triggered by the Wenchuan earthquake using a distinct element method. Environ. Earth Sci..

[52] Zhang L., Xu Y., Huang R.Q., Chang D.S. (2011). Particle flow and segregation in a giant landslide event triggered by the 2008 Wenchuan earthquake, Sichuan, China. Nat. Hazards Earth Syst. Sci..

[53] Xu Q., Fan X.-M., Huang R.-Q., Van Westen C. (2009). Landslide dams triggered by the Wenchuan Earthquake, Sichuan Province, south west China. Bull. Eng. Geol. Environ..

[54] Chigira M., Wu X., Inokuchi T., Wang G. (2010). Landslides induced by the 2008 Wenchuan earthquake, Sichuan, China. Geomorphology.

[55] Wu L., Zhu S., Wang Y., Wei K., Lu C. (2014). A modified scale method based on fractal theory for rockfill materials. Eur. J. Environ. Civ. Eng..

[56] Zhou G.G., Cui P., Tang J., Chen H., Zou Q., Sun Q. (2015). Experimental study on the triggering mechanisms and kinematic properties of large debris flows in Wenjia Gully. Eng. Geol..

[57] Zheng H., Shi Z., Peng M., Guan S., Hanley K.J., Feng S. (2022). Amplification effect of cascading breach discharge of landslide dams. Landslides.

[58] Hanson G.J., Cook K.R., Hunt S.L. (2005). Physical modeling of overtopping erosion and breach formation of cohesive embankments. Trans. Asae.

[59] Kuang S.F., Wang X.G., Huang J.C., Wei Y.Q. (2008). Risk analysis and impact assessment of dam-break in landslide lake. Chin. Water Res..

[60] Schuster R.L. (2000). A worldwide perspective on landslide dams. Usoi Landslide Dam and Lake Sarez—An Assessment of Hazard and Risk in the Pamir Mountains.

[61] Temple D.M. (1992). Estimating Flood Damage to Vegetated Deep Soil Spillways. Appl. Eng. Agric..

[62] Bennett S.J., Casalí J. (2001). Effect of initial step height on headcut development in upland concentrated flows. Water Resour. Res..

[63] Mitchener H., Torfs H. (1996). Erosion of mud/sand mixtures. Coast. Eng..

[64] Annandale G.W. (2006). Scour Technology: Mechanics and Engineering Practice.

[65] Singh V.P., Scarlatos P.D. (1988). Analysis of Gradual Earth-Dam Failure. J. Hydraul. Eng..

[66] Coleman S.E., Jack R.C., Melville B.W. Overtopping breaching of noncohesive embankment dams. Proceedings of the 27th Congress of the International Association for Hydraulic Research.

[67] Hanson G.J., Simon A. (2001). Erodibility of cohesive streambeds in the loess area of the midwestern USA. Hydrol. Process..

[68] Chen Z., Ma L., Yu S., Chen S., Zhou X., Sun P., Li X. (2015). Back Analysis of the Draining Process of the Tangjiashan Barrier Lake. J. Hydraul. Eng..

[69] Liu W., He S. (2017). Dynamic simulation of a mountain disaster chain: Landslides, barrier lakes, and outburst floods. Nat. Hazards.

[70] Garcia-Castellanos D., O’connor J.E. (2018). Outburst floods provide erodability estimates consistent with long-term landscape evolution. Sci. Rep..

[71] Chen H.X., Zhang L.M. (2015). EDDA 1.0: Integrated simulation of debris flow erosion, deposition and property changes. Geosci. Model Dev..

[72] Wu W. (2013). Simplified Physically Based Model of Earthen Embankment Breaching. J. Hydraul. Eng..

[73] Wang Z., Xu Y. (1998). A study on channel scour rate of sediment laden flow and river bed inertia. J. Sediment Res..

[74] Smerdon E.T., Beasley R.P. (1961). Critical tractive forces in cohesive soils. Agric. Eng..

[75] Amos C.L., Bergamasco A., Umgiesser G., Cappucci S., Cloutier D., DeNat L., Flindt M., Bonardi M., Cristante S. (2004). The stability of tidal flats in Venice Lagoon—the results of in-situ measurements using two benthic, annular flumes. J. Mar. Syst..

